# For the Better or for the Worse? The Effect of Manganese on the Activity of Eukaryotic DNA Polymerases

**DOI:** 10.3390/ijms25010363

**Published:** 2023-12-27

**Authors:** Eva Balint, Ildiko Unk

**Affiliations:** Institute of Genetics, HUN-REN Biological Research Centre Szeged, H-6726 Szeged, Hungary; balint.eva@brc.hu

**Keywords:** DNA polymerases, manganese, translesion synthesis, catalytic activity, polymerase families

## Abstract

DNA polymerases constitute a versatile group of enzymes that not only perform the essential task of genome duplication but also participate in various genome maintenance pathways, such as base and nucleotide excision repair, non-homologous end-joining, homologous recombination, and translesion synthesis. Polymerases catalyze DNA synthesis via the stepwise addition of deoxynucleoside monophosphates to the 3′ primer end in a partially double-stranded DNA. They require divalent metal cations coordinated by active site residues of the polymerase. Mg^2+^ is considered the likely physiological activator because of its high cellular concentration and ability to activate DNA polymerases universally. Mn^2+^ can also activate the known DNA polymerases, but in most cases, it causes a significant decrease in fidelity and/or processivity. Hence, Mn^2+^ has been considered mutagenic and irrelevant during normal cellular function. Intriguingly, a growing body of evidence indicates that Mn^2+^ can positively influence some DNA polymerases by conferring translesion synthesis activity or altering the substrate specificity. Here, we review the relevant literature focusing on the impact of Mn^2+^ on the biochemical activity of a selected set of polymerases, namely, Polβ, Polλ, and Polµ, of the X family, as well as Polι and Polη of the Y family of polymerases, where congruous data implicate the physiological relevance of Mn^2+^ in the cellular function of these enzymes.

## 1. Eukaryotic DNA Polymerases

**Figure 1:**
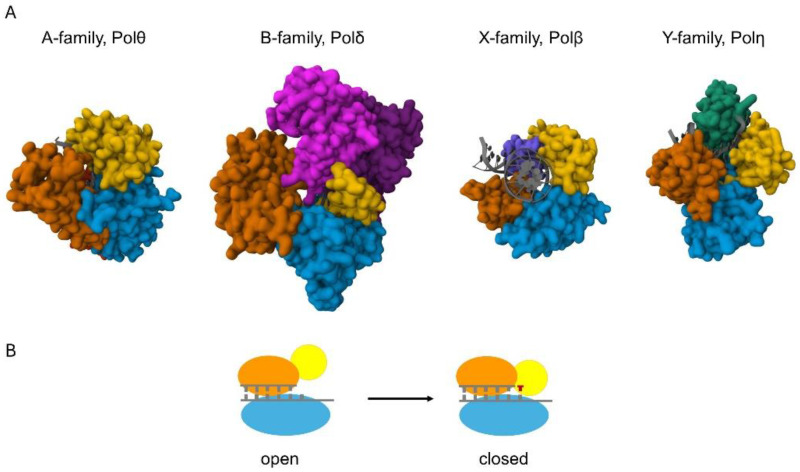
Structures of the catalytic cores of the eukaryotic DNA polymerases. (**A**) The proteins are shown in surface representation and the DNA helices are shown in cartoon representation (colored grey) [6]. The view in all the structures is down the DNA helix axis, except for Polβ, which introduces a 90° bend into the DNA. A family Pols, represented by human Polθ (4X0P) [7], possess palm (blue), fingers (yellow), thumb (orange), and exonuclease (red) domains. Exonuclease domain is positioned behind the palm and thumb domains in the figure. B family Pols, represented by yeast Polδ (3IAY) [8], have N-terminal (purple), exonuclease (magenta), palm (blue), fingers (yellow), and thumb (orange) domains. X family pols, such as human Polβ (4KLE) [9], employ palm, fingers, and thumb domains, as well as a 5′-dRP lyase domain (violet). Y family pols, represented by human Polη (3MR2) [10], have palm, fingers, and thumb domains, and possess a unique polymerase-associated domain (PAD) (green). (**B**) Schematic of the conformational change of high-fidelity polymerases. The pol binds the DNA in an “open” conformation; then, upon binding the incoming nucleotide, the finger domain (yellow) moves to a “closed” conformation that ensures correct base pairing.

DNA polymerases can synthesize DNA in a template-dependent manner [1]. They act on a primer/template DNA substrate with a free hydroxyl group at the 3′ position of the sugar moiety of the last nucleotide in the primer. The 3′-OH group is the attachment site during polymerization that proceeds through the sequential addition of dNMPs in an order directed by the template strand. One fundamental cellular task requiring this activity is genome duplication during cell division. Nevertheless, DNA synthesis is needed for several other processes, like the different DNA repair pathways, such as base (BER) and nucleotide excision repair, homologous recombination, non-homologous end-joining (NHEJ), and translesion synthesis (TLS). The structure of the substrate DNA in these pathways varies considerably. Because of this and to fulfill their cellular roles, DNA polymerases became highly specialized [2,3,4]. For example, some DNA polymerases possess exonuclease activity that proofreads mistakes committed by the enzyme. Still, others do not, while some even exhibit 5′- deoxyribophosphate (dRP) lyase, endonuclease, or terminal transferase activities. The fidelity and processivity of polymerases can also differ substantially in accordance with their cellular tasks. The diverse group of eukaryotic DNA polymerases can be classified into four families based on the primary sequence homology of the catalytic domain [5]: the A family contains pols γ, θ, and ν; the B family contains α, δ, ε, and ζ; the Y family includes η, ι, κ, and Rev1; and the X family consists of Polβ, λ, μ, and TdT. Polymerases belonging to the same family share some features, but they all exhibit individual characteristics (Figure 1).

### 1.1. B Family

The B family members are multisubunit enzymes [11]. This group includes the main replicative polymerases Polε and Polδ. Their role is to carry out faithful duplication of the genomic DNA so that the inheriting material can be transferred to the next generation of cells unchanged. In accordance with this, they are the highest-fidelity DNA polymerases. The high fidelity is attributable to their active centers that impose strict geometric selection during synthesis so that the polymerases cannot accommodate modified, damaged bases and non-Watson–Crick base pairs. In addition, pols δ and ε exhibit a 3′–5′ exonuclease activity that removes accidental errors made by the polymerases, further lowering the error rate during synthesis. Polα is a primase that provides the primer for pols δ and ε, as it can start synthesis de novo on a template strand synthesizing a short 12–15 nt RNA primer by its primase subunit, which is extended with dNTPs by its polymerase subunit. Polα does not have exonuclease activity, and because of this, its fidelity is lowered. Polζ also lacks exonuclease activity. It stands out from the group because it does not work during normal replication. It comes into play when a mismatched or damaged base introduced by other polymerases must be extended. Because its extension activity is essential for synthesis across DNA lesions, Polζ is considered a translesion synthesis polymerase, together with the Y family polymerases.

### 1.2. Y Family

Polymerases in the Y family are TLS polymerases [12]. They exhibit low fidelity on undamaged DNA due to their spacious, non-selective active center that can accommodate modified, damaged, or mismatched base pairs, in sharp contrast to replicative polymerases. Furthermore, they lack proofreading exonuclease activity. Surprisingly, they can support faithful synthesis across DNA lesions called their cognate lesions, whereas they perform error-prone synthesis across many others. These error-prone polymerases are activated when replication stalls at a DNA lesion site where a TLS polymerase replaces the replicative polymerase to carry out a lesion bypass. Beyond lesion bypass, TLS polymerases must be strictly regulated to avoid the accumulation of excess mutations in the genome. The distributive nature of their synthetic activity supports this confinement. While Pols η and ι can insert nucleotidesacross various DNA lesions, in most cases, they cannot continue the synthesis beyond the inserted nucleotide. Meanwhile, Polκ can work as an inserter and an extender during TLS due to its ability to extend from damaged or mispaired primer ends. Rev1 is very limited as a polymerase: it can catalyze the efficient incorporation of cytosine in a templated manner, whereas it is highly inefficient at inserting other nucleotides. However, it plays an essential scaffolding role during TLS by binding other TLS polymerases.

### 1.3. X Family

X family polymerases are small, gap-filling repair polymerases that function in the repair of short single-stranded DNA gaps during base excision repair, and in the direct joining of broken DNA ends with minimal or no homology during NHEJ and V(D)J recombination, which is a process that ensures immunoglobulin diversity [13]. In addition to the repair function, pols β, λ, and µ exhibit DNA lesion bypass activity. These polymerases do not have exonuclease activity, but Polβ and λ exhibit a dRP lyase activity that can remove 5′-deoxyribophosphate moieties generated by apurinic/apyrimidinic (AP) endonucleases during BER. Moreover, Polλ and µ were shown to exhibit terminal deoxynucleotidyl transferase activity, like TdT. While Polβ and λ show moderate fidelity, the error rate of Polμ is high, and TdT works primarily in a non-templated fashion and is only expressed in cells engaged in V(D)J recombination [14].

### 1.4. A Family

The A family member Polγ is responsible for the faithful duplication of the mitochondrial genome, which is supported by its high fidelity and a 3′–5′ proofreading exonuclease activity. In addition, Polγ also has a 5′-dRP lyase activity that is important for its mitochondrial repair function and it shows limited TLS capacity. In contrast, Polθ and ν are low-fidelity enzymes that take part in translesion synthesis, microhomology-mediated end joining, and DNA cross-link repair [15]. Intriguingly, Polθ shows lyase activity and it contains a helicase domain, though helicase activity has not been detected for this protein. Polν is remarkably able to bypass bulky major groove DNA adducts.

## 2. Metal Ions in DNA Polymerization

All DNA polymerases catalyze the same chemical reaction for which they apply a very similar structural arrangement of the catalytic subunit resembling a human right hand. High-fidelity polymerases undergo a conformational change during catalysis when the right-hand structure transitions from an open to a closed state (Figure 1B) [16]. This conformational change contributes to the fidelity of DNA synthesis. Keeping the analogy, the catalytic subunit has palm, thumb, and finger domains (Figure 1A). The thumb domain binds the double-stranded DNA, the fingers capture the incoming dNTP and the single-stranded template strand, and the palm contains the amino acids that coordinate two divalent metal cations essential for the chemical reaction [17]. The two metals have distinct roles and occupy different positions in the active center [18]. One serves as the catalytic metal at the so-called A site, and the other is the nucleotide metal at the B site. The A site metal helps to lower the pK_a_ of the 3′-OH proton at the primer terminus for nucleophilic attack on the α-phosphate of the incoming nucleotide. The B site metal coordinates the non-bridging oxygens of the triphosphate of the bound nucleotide and helps to neutralize the negative charge during the transition state. After a phosphodiester bond is newly formed between the 3′-O of the primer and the α-phosphate of the dNTP, a pyrophosphate is released. Several structural studies suggest the presence of a third metal during the reaction, but the exact role of the third metal is still debated [19,20]. The identity of the metal cofactor utilized by a given enzyme in the cell is usually uncertain due to technical challenges. Mg^2+^ has been considered the physiologically relevant activating metal for DNA polymerases because it is abundant in the cell and activates all known DNA polymerases in vitro. Early studies revealed that other metal ions can activate polymerases as well, but usually with much less efficiency and/or fidelity than Mg^2+^. Moreover, the cellular concentrations of other bivalent metals are significantly lower compared with Mg^2+^, supporting the pivotal role of Mg^2+^ in DNA synthesis. Particularly, the intracellular Mn^2+^ concentration is estimated to be in the µM range (up to 75 µM), whereas that of Mg^2+^ spans over to the mM range (0.2–7 mM), though the concentration is dependent on the cell type, developmental stage, and organism [21,22,23].

### Mutagenic Effect of Manganese

Like Mg^2+^, Mn^2+^ can activate all DNA polymerases. However, Cd^2+^, Co^2+^, Ni^2+^, Cr^2+^, and Mn^2+^ have been classified as mutagens and potential carcinogens because they cause polymerases to make frequent errors during DNA synthesis in vitro [24,25,26]. Mn^2+^ was shown to decrease the fidelity of viral, bacterial, and eukaryotic DNA polymerases. In the case of avian myeloblastosis virus DNA polymerase, the efficiency of DNA synthesis was half, and the misincorporation rate was twice or three times higher with Mn^2+^ compared with Mg^2+^, depending on the applied Mn concentration. Bacteriophage T4 DNA polymerase and *Escherichia coli* DNA polymerase I not only misincorporated with elevated rates but removed correctly paired nucleotides with higher rates than mispaired nucleotides if the reactions contained Mn^2+^ instead of Mg^2+^ [27]. The misinsertion capability of human DNA Polα was enhanced by Mn^2+^ as well.

## 3. Manganese Empowering DNA Polymerases

Most of the aforementioned DNA polymerases exhibit high fidelity with Mg^2+^ and are responsible for the duplication of viral, bacterial, or eukaryotic genomes. The reduced accuracy detected with Mn^2+^ would cause detrimental effects on their cellular role. However, it is clear now that beyond the deleterious effects, Mn^2+^ can improve the activity of other DNA polymerases working in DNA repair and translesion synthesis-related processes. Mn^2+^ can increase the efficiency of synthesis and the TLS capacity or change the substrate specificity of the DNA pols, enabling them to overcome a broader spectrum of obstacles. The polymerases for which substantial evidence supports Mn^2+^ in the catalytic activation are the X family members Polβ, Polλ, and Polµ, and Polι and Polη of the Y family. Below, we summarize the available data concerning the effects of Mn^2+^ on the biochemical properties of these enzymes. For comprehensive biochemical and structural summaries, we advise reading the excellent reviews published in recent years [2,3,12].

### 3.1. Polβ

Polβ is the smallest and probably the most studied eukaryotic DNA polymerase [28]. It can work on primed DNA, but its preferred substrate is a few-nucleotide gap-containing DNA where the enzyme binds both sides of the gap [29]. Polβ is considered the primary polymerase in the repair of abasic sites in BER [30]. AP sites are non-coding and, therefore, potentially mutagenic. They can arise via spontaneous hydrolysis or the action of glycosylases on modified bases. Over 10,000 AP sites are formed spontaneously in a mammalian cell in one day. Polβ catalyzes two steps of the repair where first a glycosylase removes the damaged base, leaving an AP site [31]. An AP-endonuclease makes a single-strand incision on the 5′-side of the AP site. Following this, Polβ performs high-fidelity gap-filling via its polymerase activity, using the 3′-OH at the nick, and then it removes the 5′-sugar-phosphate residues left behind by the AP endonuclease, using its 5′-dRP lyase activity. Finally, the remaining nick is sealed by a DNA ligase. Both the 5′-dRP lyase and polymerase activities are important for genomic integrity, as cancer-associated mutations exclusively affecting either the polymerase or the lyase domain were identified [28,32,33]. Moreover, Polβ is mutated in ~30% of human tumors, and disruption of the *POLB* gene coding for Polβ in mice results in embryonic lethality, which emphasizes the important role of the enzyme in maintaining genome stability [34,35,36]. During catalysis, the active center of the polymerase imposes strict geometric selection and undergoes an open-to-closed conformational transition, just like replicative polymerases [29]. This ensures accuracy, which is needed during the repair of the huge number of AP sites to preserve genome integrity. However, the fidelity is decreased compared with replicases due to the lack of intrinsic proofreading exonuclease activity. This, in turn, is advantageous for the lesion bypass activity of Polβ that is proposed to occur during gap-filling, ensuring fast repair, even in the presence of DNA lesions. 8-oxoguanine (8-oxoG), which is the major oxidative lesion [37,38], O6-methylguanine (O6mG), which is a highly mutagenic methylated lesion [39,40], N7-methylguanine (N7mG), which is the prevalent methylated lesion [41], AP-sites [42], and platinum adducts formed during chemotherapy [43,44,45,46] were shown to be bypassed by Polβ in vitro in an error-free or mutagenic manner.

Several bivalent metals, like Co^2+^, Fe^2+^, and Zn^2+^, can serve as catalysts for Polβ, but the enzyme shows the highest activity with Mg^2+^ and Mn^2+^. A wide range (0.1–10 mM) of Mg^2+^ and Mn^2+^ concentrations support catalysis without a significant loss in activity [47], though a modest decrease in fidelity is observed in the presence of Mn^2+^ (Table 1) [48,49,50]. Polβ even exhibited terminal transferase activity with Mn in crystals, which was not observed with Mg^2+^ [51]. Mn^2+^ significantly increased the bypass across an AP-site analog 3-hydroxy-2-hydroxymetylthetrahydrofuran [52]. The enzyme promoted mainly error-free synthesis opposite the major platinum adduct cisplatin-1,2-d(GpG) intramolecular crosslink with Mn^2+^ by inserting two Cs opposite the two Gs of the lesion. The misinsertion of dTMP and dATP was 100-fold less efficient [53]. Mn^2+^ enhanced the correct lesion bypass by eightfold through a fourfold decrease in the Michaelis–Menten constant (K_m_), showing the affinity of the enzyme to the substrate, and a twofold increase in the velocity of the reaction (k_cat_) [54]. Similarly, Polβ could catalyze the accurate bypass across an N7mG analog 2′-fluoro-m7dG in a gapped substrate with Mg^2+^ via inserting the correct C, and no misinsertion was observed with T, even with Mn^2+^ [41]. During the bypass of O6mG with Mg^2+^, Polβ inserted the incorrect T with almost 20-fold higher efficiency than C opposite the lesion [40,54,55]. Using Mn^2+^, the efficiency of misincorporation became 10-fold higher due to increased velocity, whereas the insertion efficiency of the correct C was twofold higher compared with Mn^2+^, resulting in lowered fidelity [55]. Polβ could synthesize across thymin glycol, which is the most common oxidation product of thymine, using all four dNTPs in the presence of Mg^2+^, inserting the correct A 10–100-fold more efficiently than an incorrect nucleotide [56]. Mn^2+^ enhanced error-free insertion but also the misinsertion of non-complementary nucleotides, mainly through a 100-fold decrease in K_m_. Based on the above examples, it seems that Mn^2+^ does not alter the nucleotide preference of Polβ but it makes the enzyme more active and the bypass more efficient. Even though the enhancement is modest in most cases, it can have a significant impact since lesion bypassing by Polβ is highly inefficient with Mg^2+^. For example, Polη bypasses platinum adducts with 40% efficiency, whereas Polβ with 2% efficiency of the synthesis on the unmodified template, and the synthesis opposite O6mG is 100-fold less efficient and across 2′-fluoro-m7dG is 300-fold less efficient than on undamaged DNA [40,41,44,57].

### 3.2. Polλ

Polλ exhibits all the catalytic activities possessed by other members of the X family [58,59]. It has polymerase, terminal transferase, polynucleotide synthetase, and 5′-dRP lyase, but lacks exonuclease activity [60,61,62]. It exhibits a 34% similarity of the catalytic domain with Polβ at the amino acid level. Despite this, the catalytic domain of Polλ does not undergo a large open-to-closed conformation change during catalysis, as opposed to Polβ [63]. Rather, it stays in a conformation resembling the closed state, even without substrate binding. The lack of a substrate-binding induced large conformational shift of the catalytic domain contributes to the lowered selectivity of the active center of Polλ compared with Polβ. It can even accommodate large G:G mispairs [64]. Polλ misinserts nucleotides during synthesis with a ~10-fold lower fidelity than Polβ, partly due to its ability to prebind nucleotides, even in the absence of DNA [65]. It generates single base deletions at a high rate caused by primer/template misalignment [66]. Synthesis by Polλ is distributive on a template/primer substrate but processive during the filling of short gaps [67]. These features suggest a more versatile role for Polλ compared with Polβ. Utilizing its activities, Polλ participates in BER, NHEJ, and V(D)J recombination. In BER, it can substitute for Polβ, and in NHEJ and V(D)J recombination, Polλ can fill the small gaps generated by the alignment of broken DNA ends having at least one base pair microhomology [68,69,70,71]. During gap filling, Polλ can perform translesion synthesis by inserting across lesions and it can extend from damaged DNA ends. The enzyme was shown to promote the error-prone bypass of AP sites [72], as well as the error-free bypass of 8-oxoG by preferentially extending the correct nucleotide opposite the lesion [73,74,75], 2-hydroxy adenine [76], (6-4)TT photoproducts [77], and N1-methyl-deoxyadenosine [78].

Interestingly, Polλ prefers Mn^2+^ as a catalytic cofactor in in vitro experiments. It showed a threefold higher activity on a primer/template at a close-to-physiological (lower than 1 mM) Mn^2+^ concentration but was inhibited at physiological (above 2 mM) Mg^2+^ concentrations (Table 2) [47]. In contrast, Polα and Polδ were still active at 10–30 mM Mg^2+^ and inhibited at 1 mM or higher Mn^2+^. On primer/template substrates insertions of the correct and incorrect dNMPs were 1.5- and 5-fold more efficient with Mn^2+^, respectively, yielding a slight decrease in fidelity [79]. Polλ inserted rNMPs into a primer/template with 100–200-fold less efficiency than dNMPs with Mg^2+^, and this activity was increased 5–25-fold with Mn^2+^. As opposed to a primer/template, Mn^2+^ conferred a 100-fold increase in activity on a gapped DNA representing the cognate substrate of Polλ in the cell [56]. Polλ also showed higher TLS capacity in the presence of Mn^2+^ as the error-free bypass of a thymine glycol in gapped substrates was 30-fold, and the misinsertion of dGMP was 60-fold more efficient compared with Mg^2+^, resulting in a mere twofold decrease in fidelity [56]. Mn^2+^ enabled Polλ to bypass an AP site [52]. The available data are too scarce to draw a definite conclusion, but the results show that similarly to Polβ, Mn^2+^ elevates the activity of Polλ on a gapped substrate, damaged or undamaged, without compromising the insertion preference of the enzyme.

### 3.3. Polµ

Polµ is a low-fidelity, distributive polymerase that functions in NHEJ and the V(D)J recombination [71,80,81,82,83]. The enzyme shares 41% identity with TdT at the amino acid level and besides the template-dependent polymerase activity, it exhibits template-independent terminal transferase activity [84]. It possesses several surprising features not found with other polymerases. It is a versatile enzyme that can act on various substrates in vitro, like gap-containing templates, unpaired primers, or overhanging primers [71]. Moreover, Polμ stands out from the other polymerases by having the ability to align broken DNA ends that have no complementarity at all. Its terminal transferase activity, which involves extending single-stranded DNA, was proposed to be required for creating or increasing the complementarity of broken DNA ends [85]. During the repair of small gaps, Polµ can realign the primer/template so that it skips the first templating nucleotide and uses the nucleotide adjacent to the 5′ side of the gap instead as a template generating a few nucleotide deletions with high frequency [86,87]. The enzyme remains rigid throughout the catalytic cycle without showing even the small dNTP-binding-induced movements of active site side chains characteristic of Polλ [88]. This rigidity is probably required to firmly engage the often unstable DNA substrates of NHEJ. During template-dependent synthesis, Polµ can utilize both dNTPs and rNTPs with almost the same efficiency [89,90,91]. It discriminates between rNTPs and dNTPs with a 1000-fold lower efficiency than Polβ, showing a mere 10-fold or lower preference toward dNTPs. In addition, Polµ exhibits lesion bypass activity opposite several DNA lesions. It bypasses most of the lesions, such as AP-site, 8-oxoG, platinum adducts, and other bulky lesions, in an error-prone way by realigning the primer/template [92,93,94]. Surprisingly, during the bypass of the UV-induced TT dimer, Polµ primarily inserts the two correct As opposite the lesion [92].

Like its siblings in the X family, Polµ is more active with Mn^2+^ compared with Mg^2+^ [84]. Mn^2+^ was suggested to be the in vivo metal co-factor of Polμ since it promoted efficient insertion of only the correct nucleotide at physiological concentrations (10–40 μM), whereas a high concentration (1 mM) compromised the fidelity of Polμ on various gapped substrates [95]. Fidelity was further increased when rNTPs were added instead of ddNTPs. Although the kinetic parameters were not determined, a similar amount of insertion products were obtained using 100-fold lower concentrations of ddNTPs or rNTPs in the presence of Mn^2+^ versus Mg^2+^. When physiological concentrations of nucleotides were provided, an up to 30-fold shorter time was needed to obtain the same amount of insertion product with Mn^2+^ compared with Mg^2+^. Polμ could also extend RNA primers with rNTPs and Mn^2+^ strongly improved the incorporation [89]. Single-turnover kinetic analysis and time-lapse X-ray crystallography using a gapped substrate and dNTPs showed that the overall insertion efficiency (k_pol_/K_d_) was 50-fold higher in the presence of Mn^2+^ versus Mg^2+^. This increase resulted mostly from the enhanced velocity, as the rate constant (k_pol_) of Polμ was 13.5 fold higher, while the K_d_ for the templated correct incoming dNTP was only 3.5-fold lower (Table 3) [96]. Mn^2+^ enhanced the incorporation of both 8-oxo-dGTP and 8-oxo-rGTP into a gapped substrate [97,98].

Like Polλ, Polµ can prebind the nucleotide before binding the template DNA [51,100]. In the presence of Mg^2+^, Polµ prebinds dNTPs or rNTPs with low affinity, with K_d_ values in the range of 98−236 μM, as opposed to Mn^2+^, which promotes much stronger prebinding with K_d_ values in the range of 0.22−2.76 μM. Although nucleotide prebinding before binding the DNA template causes infidelity, it can be very advantageous for creative, non-templated synthesis. Based on its preference in vitro, it was proposed that rNTPs and Mn^2+^ are the physiological substrates of Polμ during NHEJ. Indeed, 65% of cellular NHEJ products contained transiently embedded rNTPs, which was dependent on Polμ [101]. Additionally, NHEJ reconstitution experiments with Polμ showed that Mn^2+^ and rNTP insertion stimulated the coupled ligation of the broken DNA strands, whereas, with Mg^2+^ and dNTPs, the ligation step was inefficient [102].

### 3.4. Polι

Polι belongs to the Y family of TLS polymerases, which rescue stalled replication forks by synthesizing across DNA lesions or bypassing DNA damage during postreplication repair [103]. Uniquely among TLS polymerases, Polι has an additional 5′-dRP lyase activity through which it can participate in BER [104]. It is a very distributive and error-prone polymerase; however, its infidelity is strongly sequence-dependent and may differ by 100,000-fold [105,106,107]. Polι is the most accurate opposite template A; it is less accurate opposite templates G or C; while fidelity is completely lost opposite template T, where Polι favors the incorporation of a wrong G instead of the correct A. The crystal structure of the catalytic domain revealed that unlike other polymerases, Polι binds the template base in a flipped “syn” conformation instead of the usual “anti” conformation because of its narrow active site [108]. This results in the formation of Hoogsteen hydrogen bonds with the incoming nucleotide instead of Watson–Crick base pairing. Moreover, in the case of template T, Polι may induce wobble base pairing with the incoming nucleotide [109]. This versatility of Polι enables the enzyme to efficiently bypass various types of DNA damage, like AP-sites, 8-oxoG, CPD, 6–4 photoproducts, and methyl adducts [110,111,112,113,114,115].

Polι exhibited the greatest activity in the presence of Mn^2+^ in in vitro primer extension experiments [116]. The enzyme showed the highest activity in low, close-to-physiological concentrations of Mn^2+^ (50–250 μM) and low levels of Mg^2+^ (100–500 µM), whereas Mg^2+^ concentrations higher than 2 mM had a strong inhibitory effect. The enzyme preferred Mn^2+^, even in a 40-fold excess of Mg^2+^. Steady-state kinetics analysis showed that Polι was 30–6000-fold more active in the presence of 75 μM Mn^2+^ than in the presence of 5 mM Mg^2+^ (Table 4). The increase was the result of a huge decrease in K_m_, while the v_max_ values were virtually unchanged. When applying low 250 µM Mg^2+^, the change was less dramatic. However, interesting individual differences were observed on various templates. Opposite template T, the K_m_ value for the correct incorporation of A decreased by 10-fold, while for the incorrect G, it decreased fivefold in the presence of low Mn^2+^ versus low Mg^2+^. This led to an increase in the fidelity of the enzyme now favoring the incorporation of the correct A by twofold instead of the incorrect G. In contrast, on template A, the K_m_ value decreased by 30-fold for the correct incorporation of T and 23,000-fold for the incorrect A when using Mn^2+^ versus Mg^2+^. This resulted in a steep fall in fidelity with the preference for correct T dropping from 4000-fold to 13-fold. Thus, Mn^2+^ increased the polymerization activity of Polι on both templates but it increased the fidelity on template T while decreasing it on template A. Polι could incorporate rNTPs during primer extension with Mn^2+^ [117]. Its poor base selectivity observed for dNTPs was improved when using rNTPs. Furthermore, Mn^2+^ enhanced the DNA damage bypass ability of Polι opposite several lesions such as CPD, 6–4 photoproduct, BPDE, AP-site, and N2-ethylamine [116,118]. In summary, Mn^2+^ improved the catalytic efficiency of Polι on undamaged DNA, concomitantly altering its nucleotide preference, and hence the fidelity of the enzyme, and it increased the efficiency of TLS as well. The relevance of its strange biochemical characteristics and the exact function of Polι are still enigmatic. It was proposed to take part in the somatic hypermutation of immunoglobulin genes, but unambiguous in vivo data are still lacking [119,120,121,122]. On the other hand, the role of Polι in the cellular response to different DNA-damaging agents is supported by several studies [115,123,124]. It contributed to the UV resistance and UV-induced mutagenesis of human cells and the mutagenic replication through the anticancer nucleoside cytarabine.

### 3.5. Polη

Like the other Y-family polymerases, Polη has low fidelity and low processivity [125,126]. Despite this, its inactivity in humans causes a UV-induced cancer-prone syndrome, which is the variant form of xeroderma pigmentosum [127]. This suggests that the major physiological function of Polη is the non-mutagenic bypass of UV lesions. Indeed, Polη has the unique ability to efficiently and error-freely synthesize opposite CPD, which is the major UV-induced lesion [128,129]. The crystal structures of the catalytic domain of yeast and human Polη revealed that Polη has a more spacious active center than other polymerases and can accommodate cross-linked or bulky damaged bases [10,130,131]. Furthermore, structures with undamaged and damaged nucleotides are superimposable, meaning that the active site geometry is not disturbed by the lesion. This is because the catalytic domain is rigid; it acts as a molecular “splint” holding the DNA containing the lesion firmly in a stable conformation to allow for the correct base pairing and ensuing new chemical bond formation. Accordingly, Polη was shown to perform error-free or error-prone bypassing of a plethora of lesions, including 8-oxoG, [132], other oxidative damage, such as cdA [133] and 8-oxoadenine [134], AP-sites [135,136], alkylated nucleotides, such as O6mG [137,138], N7mG [48,139], N7-nitrogen half-mustard (NHMG) [140], N7-benzylguanine [141], and deaminated purines, like xanthine and hypoxanthine [140], as well as platinum adducts resulting from chemotherapy [142,143,144] and cross-linked peptides [145]. Additionally, Polη was shown to accommodate RNA strands for catalysis, either as a template for performing reverse transcription or as a primer for synthesizing polyribonucleotide chains or making mixed RNA-DNA strands [123,146,147,148,149,150]. Moreover, Polη could insert rNTPs during lesion bypassing [151]. However, rNTP incorporation by Polη is very inefficient with Mg^2+^.

Besides Mg^2+^, Polη can also be activated by Mn^2+^ during DNA synthesis but with lower efficiency and with the price of losing base selectivity. Surprisingly, the RNA synthetic activity of Polη is greatly enhanced by Mn^2+^ without significantly affecting its fidelity (Table 5) [152,153].

The optimal Mn^2+^ concentration for Polη during RNA synthesis is about 5 mM, which is much higher than the physiological Mn^2+^ concentration, though reduced activity could be observed below 1 mM Mn^2+^. In the case of yeast Polη, Mn^2+^ enhanced the incorporation of the correct rNTP 400 to 2000-fold. This increase in catalytic efficiency was due to a 2–30-fold increase in the k_cat_ and a 40–250-fold decrease in K_m_. Importantly, the incorporation of the correct rNTPs opposite a TT dimer or 8-oxoG, which are the cognate DNA lesions of Polη, were stimulated 3000- and 6000-fold, respectively, highlighting that Mn^2+^ specifically enhanced the damage bypass ability of Polη during RNA synthesis. Incorporation of the incorrect nucleotide was also enhanced somewhat, but the misincorporation frequency remained in the range of the 10^−1^ to 10^−4^ observed during DNA synthesis; therefore, fidelity was maintained. Similar results were obtained with human Polη, where Mn^2+^ increased the efficiency of incorporation of the correct rNTPs 200 to 1250-fold and the correct bypass of a TT dimer and 8-oxoG 200-fold compared with Mg^2+^ (Table 6) [153]. The increase was mainly due to a decreased K_m_, while the k_cat_ change was negligible. The misincorporation frequency did not change on undamaged templates and across 8-oxoG. Notably, error-free synthesis across a TT dimer became threefold more efficient than the opposite undamaged T with Mn^2+^, but the fidelity was somewhat lowered. During DNA synthesis, Mn^2+^ enabled Polη to replicate through difficult-to-bypass lesions. For example, in the presence of Mg^2+^, the synthesis opposite cdA, which is a rigid oxidative lesion, was extremely weak, with the k_cat_/K_m_ being 0.015 min^−1^ μM^−1^, whilst, in the presence of Mn^2+^, it almost equaled the efficiency observed opposite the undamaged A [133]. Mn^2+^ enhanced the incorporation opposite the undamaged template ninefold, while it enhanced the bypass of the damage 1400-fold. This enhancement resulted from a similar increase in the affinity toward the incoming nucleotide, while the turnover rate did not change appreciably. At the same time, only the incorporation of the correct dTTP was detected, thus Mn^2+^ did not compromise the fidelity of Polη. Similarly, opposite a frequent alkylated purine N7-benzylguanine (N7BnG), Mn^2+^ enhanced the incorporation of the correct dCTP threefold, whereas that of the incorrect dTTP was increased fivefold; hence, the fidelity was somewhat lowered [141]. In conclusion, on one hand, Mn^2+^ decreased the activity of Polη and compromised its fidelity on undamaged DNA using dNTPs, whereas it enhanced its damage bypass ability. On the other hand, it greatly increased the RNA synthetic activity of the enzyme without affecting its fidelity on undamaged DNA and promoted an even greater increase in the TLS activity during RNA synthesis.

Though the roles of Polη in HR, damage-induced sister chromatid cohesion, and transcription were described, its primary function is in the bypass of DNA lesions [154]. Mn^2+^ as a metal cofactor expands the TLS activity of Polη from DNA synthesis to RNA synthesis as well.

## 4. Conclusions and Future Perspectives

A small number of different DNA polymerases are available for a cell to cope with DNA lesions and DNA structure alterations that are extremely high in variety and number. Each of these so-called repair or TLS polymerases is adept at handling numerous different challenging structures, but their abilities are limited. A growing body of evidence indicates that these polymerases increase their versatility by replacing their metal cofactor Mg^2+^ with Mn^2+^. Mn^2+^ can change the biochemical characteristics of polymerases. It can elevate or decrease the normal activity, enable lesion bypass, and change fidelity or substrate specificity; all this is undertaken to better suit the task of genome maintenance. The examples discussed above suggest that besides Mg^2+^, Mn^2+^ can work as a physiological metal cofactor for DNA polymerases, though supporting in vivo studies are scarce. Y and X family polymerases are not the only targets of Mn^2+^. Mn^2+^ was shown to enhance the activity of PrimPol, which is a recently discovered primase polymerase that belongs to the family of archaic primases and may play a role in re-priming synthesis after DNA damage (Tokarsky 2017). Furthermore, Mn^2+^ is the cofactor of Mre11, which is a nuclease functioning in HR. To further support the role of Mn^2+^ in DNA polymerase activation, more kinetic and thermodynamic data, as well as state-of-the-art biophysical methods, are required besides in vivo experiment. The interesting question of how the different metal cofactors are acquired by DNA polymerases remains to be answered, as well.

## Figures and Tables

**Table 1:** Comparison of the effect of Mg^2+^ and Mn^2+^ on the catalytic activity and fidelity of Polβ.

Substrate, Templating Nucleotide	Incoming Nucleotide	Cation	Velocity Constant	Affinity Constant	Efficiency	Magnitude of Stimulation, Mn^2+^/Mg^2+ a^	Misinsertion Frequency ^b^	Reference
1 nt gapped DNA, G			k_cat_ (10^−3^ s^−1^) ^c^	K_m_ (μM) ^c^	k_cat_/K_m_ (10^−3^ s^−1^ μM^−1^)			[48]
	dCTP	5 mM Mg^2+^	212.0 ± 19.9	0.6 ± 0.1	353.3	1	1	
	dCTP	5 mM Mn^2+^	30.3 ± 1.5	0.08 ± 0.01	383.7	1.08	1	
	dTTP	5 mM Mg^2+^	2.8 ± 0.4	56.1 ± 4.6	0.049	1	1.4 × 10^−4^	
	dTTP	5 mM Mn^2+^	19.1 ± 0.8	11.2 ± 0.5	1.71	34	4.5 × 10^−3^	
1 nt gapped DNA, O^6^MedG	dCTP	5 mM Mg^2+^	14.5 ± 1.2	234.2 ± 24.5	0.062	1	1	[55]
	dCTP	5 mM Mn^2+^	20.4 ± 1.6	193.3 ± 7.6	0.11	1.7	1	
	dTTP	5 mM Mg^2+^	62.4 ± 11.0	56.2 ± 4.7	1.1	1	17	
	dTTP	5 mM Mn^2+^	431.8 ± 53.2	38.7 ± 4.1	11.2	10	100	
1 nt gapped DNA, Pt-GG	dCTP	5 mM Mg^2+^	15.76 ± 1.24	5.22 ± 1.01	3.0	1		[54]
	dCTP	5 mM Mn^2+^	27.60 ± 1.62	1.14 ± 0.05	24.2	8		
			k_cat_ (s^−1^)	K_m_ (μM)	k_cat_/K_m_ (μM^−1^ s^−1^)			[56]
5 nt gapped DNA, T	dATP	1 mM Mg^2+^	0.084	0.019	4.4	1		
	dATP	1 mM Mn^2+^	0.078	0.085	0.92	0.2		
5 nt gapped DNA, Thymine glycol	dATP	1 mM Mg^2+^	0.038	11.3	0.0034	1	1	
	dATP	1 mM Mn^2+^	0.084	0.07	1.2	360	1	
	dGTP	1 mM Mg^2+^	0.0064	12.5	0.0005	1	1.5 × 10^−1^	
	dGTP	1 mM Mn^2+^	0.064	3.51	0.018	36	1.5 × 10^−2^	
2 nt gapped DNA, Thymine glycol	dATP	1 mM Mg^2+^	0.068	0.322	0.21	1	1	
	dATP	1 mM Mn^2+^	0.093	0.043	2.2	10	1	
	dGTP	1 mM Mg^2+^	0.0073	0.162	0.045	1	2.1 × 10^−1^	
	dGTP	1 mM Mn^2+^	0.083	0.653	0.13	2.8	6 × 10^−2^	
1 nt gapped DNA, Thymine glycol	dATP	1 mM Mg^2+^	0.090	0.371	0.24	1	1	
	dATP	1 mM Mn^2+^	0.092	0.006	15	63	1	
	dGTP	1 mM Mg^2+^	0.070	61.73	0.0011	1	4.7 × 10^−3^	
	dGTP	1 mM Mn^2+^	0.076	0.337	0.23	200	1.4 × 10^−2^	

^a^ Magnitude of stimulation by Manganese was calculated as relative efficiency: f_rel_ = (k_cat_/K_m_)_Mn^2+^_/(k_cat_/K_m_)_Mg^2+^_. ^b^ Misinsertion frequency was calculated as relative efficiency: f_rel_ = (k_cat_/K_m_)_incorrect_/(k_cat_/K_m_)_correct_. ^c^ Steady-state kinetics: k_cat_ is the turnover rate of the enzyme and K_m_ is the Michaelis–Menten constant. Rows showing data measured in the presence of Mn^2+^ have a blue background.

**Table 2:** Comparison of the effect of Mg^2+^ and Mn^2+^ on the catalytic activity and fidelity of Polλ.

Substrate (Primer/Template), Templating Nucleotide	Incoming Nucleotide	Cation	Velocity Constant	Affinity Constant	Efficiency	Magnitude of Stimulation, Mn^2+^/Mg^2+ a^	Misinsertion Frequency ^b^	Reference
			k_cat_ (s^−1^) ^c^	K_m_ (μM) ^c^	k_cat_/K_m_ (s^−1^ M^−1^) ^c^			[47]
oligo(dT)/poly(dA), A	dTTP	1 mM Mg^2+^	0.006	4.7	1.2 × 10^3^	1		
	dTTP	1 mM Mn^2+^	0.016	3.2	5 × 10^3^	4		
			k_cat_ (min^−1^) ^d^	K_d_ (μM) ^d^	k_cat_/K_d_ (min^−1^ mM^−1^) ^b^			
19/40-mer, T	dATP	1 mM Mg^2+^	0.05 ± 0.01	0.8 ± 0.1	0.0625 ± 0.01	1	1	[79]
	dATP	0.1 mM Mn^2+^	0.12 ± 0.02	1.2 ± 0.1	0.1 ± 0.05	1.6	1	
	dCTP	1 mM Mg^2+^	0.002 ± 0.0005	4.5 ± 0.5	0.0004 ± 0.0001	1	6.4 × 10^−3^	
	dCTP	0.1 mM Mn^2+^	0.01 ± 0.003	4.5 ± 0.5	0.0022 ± 0.0002	5.5	2.2 × 10^−2^	
	dGTP	1 mM Mg^2+^	0.008 ± 0.004	3 ± 0.3	0.002 ± 0.0003	1	3.2 × 10^−2^	
	dGTP	0.1 mM Mn^2+^	0.02 ± 0.01	2.5 ± 0.2	0.008 ± 0.01	4	8.0 × 10^−2^	
	ATP	1 mM Mg^2+^	0.01 ± 0.002	12 ± 2	0.0008 ± 0.0001	1	1.3 × 10^−2^	
	ATP	0.1 mM Mn^2+^	0.015 ± 0.005	3.7 ± 2	0.004 ± 0.001	5	4.0 × 10^−2^	
20/40-mer, G	dCTP	1 mM Mg^2+^	0.2 ± 0.08	0.9 ± 0.1	0.22 ± 0.03	1	1	[79]
	dCTP	0.1 mM Mn^2+^	0.5 ± 0.2	1.5 ± 0.1	0.33 ± 0.04	1.5	1	
	dGTP	1 mM Mg^2+^	0.004 ± 0.001	0.8 ± 0.2	0.005 ± 0.001	1	2.3 × 10^−2^	
	dGTP	0.1 mM Mn^2+^	0.04 ± 0.003	1.4 ± 0.1	0.028 ± 0.006	5.6	8.5 × 10^−2^	
	CTP	1 mM Mg^2+^	0.01 ± 0.003	9 ± 2	0.0011 ± 0.0002	1	5 × 10^−3^	
	CTP	0.1 mM Mn^2+^	0.08 ± 0.01	2.7 ± 0.5	0.029 ± 0.004	26	8.8 × 10^−3^	
21/40-mer, C	dGTP	1 mM Mg^2+^	0.08 ± 0.01	3 ± 0.4	0.026 ± 0.002	1	1	[79]
	dGTP	0.1 mM Mn^2+^	0.2 ± 0.03	2.5 ± 0.2	0.08 ± 0.02	3	1	
	GTP	1 mM Mg^2+^	0.003 ± 0.001	10 ± 1	0.0003 ± 0.0001	1	1.1 × 10^−2^	
	GTP	0.1 mM Mn^2+^	0.03 ± 0.01	6.5 ± 0.7	0.0046 ± 0.001	15	5.8 × 10^−2^	
			k_cat_ (s^−1^) ^c^	K_m_ (μM) ^c^	k_cat_/K_m_ (μM^−1^ s^−1^)			[56]
5 nt gapped DNA, T	dATP	Mg^2+^	0.026	6.44	0.0041	1		
	dATP	Mn^2+^	0.060	0.144	0.42	100		
2 nt gapped DNA, Thymineglycol	dATP	Mg^2+^	0.006	0.091	0.069	1	1	
	dATP	Mn^2+^	0.027	0.012	2.3	34	1	
	dGTP	Mg^2+^	0.011	1.42	0.008	1	1.2 × 10^−1^	
	dGTP	Mn^2+^	0.043	0.083	0.52	67	2.3 × 10^−1^	

^a^ Magnitude of stimulaton by Manganese was calculated as relative efficiency: f_rel_ = (k_cat_/K_m_)_Mn^2+^_/(k_cat_/K_m_)_Mg^2+^_. ^b^ Misinsertion frequency was calculated as relative efficiency: f_rel_ = (k_cat_/K_m_)_incorrect_/(k_cat_/K_m_)_correct_. ^c^ Steady-state kinetics: kcat is the turnover rate of the enzyme and K_m_ is the Michaelis–Menten constant. ^d^ Pre-steady-state kinetics: k_pol_ is the maximum rate constant of the pre-steady-state burst phase and K_d_ is the dissociation constant of the Pol–DNA–dNTP complex. Rows showing data measured in the presence of Mn^2+^ have a blue background.

**Table 3:** Comparison of the effects of Mg^2+^ and Mn^2+^ on the catalytic activity and fidelity of Polµ.

Substrate, Templating Nucleotide	Incoming Nucleotide	Cation	Velocity Constant	Affinity Constant	Efficiency	Magnitude of Stimulation, Mn^2+^/Mg^2+ a^	Misinsertion Frequency ^b^	Reference
1 nt gapped DNA, A			k_pol_ (s^−1^) ^d^	K_d_ (μM) ^d^	k_pol_/K_d_ (μM^−1^ s^−1^) ^c^			[96]
	dTTP	10 mM Mg^2+^	6.0 ± 0.2	192 ± 21	0.031 ± 0.004	1		
	dTTP	1 mM Mn^2+^	81 ± 7	54 ± 18	1.5 ± 0.5	48		
			k_cat_ (min^−1^) ^c^	K_m_ (μM) ^c^	k_cat_/K_m_ (min^−1^ μM^−1^)			[98]
1 nt gapped DNA, C	dGTP	10 mM Mg^2+^	5.70 ± 0.21	3.48 ± 0.31	1.64 ± 0.16	1	1	
	dGTP	1 mM Mn^2+^	0.16 ± 0.01	0.006 ± 0.001	26.9 ± 4.7	16	1	
1 nt gapped DNA, A	dGTP	10 mM Mg^2+^	0.04 ± 0.01	55.6 ± 5.9	0.0007 ± 0.0002	1	4.3 × 10^−4 e^	
	dGTP	1 mM Mn^2+^	2.98 ± 0.01	21.3 ± 2.3	0.14 ± 0.02	200	5.2 × 10^−3 e^	
1 nt gapped DNA, T			k_cat_ (10^−3^ min^−1^) ^c^	K_m_ (μM) ^c^	k_cat_/K_m_ (min^−1^ μM^−1^)			[99]
	dATP	10 mM Mg^2+^	236.2 ± 5.3	3.1 ± 0.3	76.6 × 10^−3^			
	dGTP	10 mM Mg^2+^	28.3 ± 0.6	5.7 ± 0.5	4.9 × 10^−3^	1	6.4 × 10^−2^	
	dGTP	10 mM Mn^2+^	100.1 ± 2.6	5.2 ± 0.6	19.2 × 10^−3^	3.9	n.d.	

^a^ Magnitude of stimulaton by Manganese was calculated as relative efficiency: f_rel_ = (k_cat_/K_m_)_Mn^2+^_/(k_cat_/K_m_)_Mg^2+^_. ^b^ Misinsertion frequency was calculated as relative efficiency: f_rel_ = (k_cat_/K_m_)_incorrect_/(k_cat_/K_m_)_correct_.^c^ Steady-state kinetics: k_cat_ is the turnover rate of the enzyme and K_m_ is the Michaelis–Menten constant. ^d^ Pre-steady-state kinetics: k_pol_ is the maximum rate constant of the pre-steady-state burst phase and K_d_ is the dissociation constant of the Pol–DNA–dNTP complex. ^e^ Unconventionally, here, the same incoming nucleotide was compared opposite different templating bases. Rows showing data measured in the presence of Mn^2+^ have a blue background.

**Table 4:** Comparison of the effect of Mg^2+^ and Mn^2+^ on the catalytic activity and fidelity of Polι.

Substrate (Primer/Template), Templating Nucleotide	Incoming Nucleotide	Cation	Velocity Constant	Affinity Constant	Efficiency	Magnitude of Stimulation, Mn^2+^/Mg^2+ a^	Misinsertion Frequency ^b^	Reference
			V_max_ (% min^−1^) ^c^	K_m_ (μM) ^c^	V_max_/K_m_ (% min^−1^ μM^−1^)			[116]
16/48-mer, T	dATP	5 mM Mg^2+^	5 ± 0.8	2.7 ± 0.5	1.85	1	1	
	dATP	0.075 mM Mn^2+^	7.1 ± 0.5	0.0011 ± 0.0002	6450	3486	1	
	dGTP	5 mM Mg^2+^	8.3 ± 0.9	1.8 ± 0.3	4.6	1	2.5	
	dGTP	0.075 mM Mn^2+^	6.3 ± 1	0.0022 ± 0.0003	2860	622	4.4 × 10^−1^	
20/50-mer, A	dTTP	5 mM Mg^2+^	10 ± 2	0.06 ± 0.01	167	1	1	
	dTTP	0.075 mM Mn^2+^	2.6 ± 0.5	0.00053 ± 0.0001	4900	30	1	
	dATP	5 mM Mg^2+^	5 ± 1.1	90 ± 17	0.06	1	3.6 × 10^−4^	
	dATP	0.075 mM Mn^2+^	1.1 ± 0.3	0.003 ± 0.0008	370	6600	7.5 × 10^−2^	
			k_cat_ (min^−1^) ^c^	K_m_ (μM)^c^	k_cat_/K_m_ (min^−1^ μM^−1^)			[118]
14/32-mer, G	dCTP	2 mM Mg^2+^	112 ± 18	49 ± 4	2.3	1	1	
	dCTP	0.075 mM Mn^2+^	700 ± 40	0.15 ± 0.020	4700	2040	1	
	dTTP	2 mM Mg^2+^	50 ± 6	220 ± 60	0.23	1	1.0 × 10^−1^	
	dTTP	0.075 mM Mn^2+^	200 ± 15	0.085 ± 0.020	2350	10,000	5.0 × 10^−1^	
14/32-mer, N2-ethyl-G	dCTP	2 mM Mg^2+^	74 ± 12	36 ±3	2.1	1	1	
	dCTP	0.075 mM Mn^2+^	425 ± 15	0.10 ± 0.012	4250	2020	1	
	dTTP	2 mM Mg^2+^	115 ± 18	650 ± 180	0.18	1	8.6 × 10^−2^	
	dTTP	0.075 mM Mn^2+^	225 ± 25	0.030 ± 0.014	7500	41,700	1.7	

^a^ Magnitude of stimulaton by Manganese was calculated as relative efficiency: f_rel_ = (k_cat_/K_m_)_Mn^2+^_/(k_cat_/K_m_)_Mg^2+^_. ^b^ Misinsertion frequency was calculated as relative efficiency: f_rel_ = (k_cat_/K_m_)_incorrect_/(k_cat_/K_m_)_correct_. ^c^ Steady-state kinetics: k_cat_ is the turnover rate of the enzyme, K_m_ is the Michaelis–Menten constant, and V_max_ is maximum velocity of the reaction expressed as percentage of primer extended. Rows showing data measured in the presence of Mn^2+^ have a blue background.

**Table 5:** Comparison of the effect of Mg^2+^ and Mn^2+^ on the catalytic activity and fidelity of yeast Polη.

Substrate (Primer/Template), Templating Nucleotide	Incoming Nucleotide	Cation	Velocity Constant	Affinity Constant	Efficiency	Magnitude of Stimulation, Mn^2+^/Mg^2+ a^	Misinsertion Frequency ^b^	Reference
			k_cat_ (min^−1^) ^c^	K_m_ (µM) ^c^	k_cat_/K_m_ (min^−1^ µM^−1^)			[152]
30-mer RNA/50-mer DNA, T	rATP	5 mM Mg^2+^	0.24 ± 0.01	466± 47.3	5.15 × 10^−4^	1		
	rATP	5 mM Mn^2+^	2.61 ± 0.14	2.51 ± 0.64	1.04	2019	1	
	rCTP	5 mM Mn^2+^	1.72 ± 0.06	19.4 ± 3.14	9 × 10^−2^		8.7 × 10^−4^	
30-mer RNA/50-mer DNA, G	rCTP	5 mM Mg^2+^	2.76 ± 0.06	438 ± 37.5	6.30 × 10^−3^	1		
	rCTP	5 mM Mn^2+^	4.68 ± 0.22	1.89 ± 0.42	2.48	394	1	
	rGTP	5 mM Mn^2+^	0.31 ± 0.02	68.9 ± 14.9	4.5 × 10^−3^		1.8 × 10^−3^	
30-mer RNA/50-mer DNA, C	rGTP	5 mM Mg^2+^	0.45 ± 0.01	394 ± 52	1.14 × 10^−3^	1		
	rGTP	5 mM Mn^2+^	5.07 ± 0.27	2.55 ± 0.63	1.99	1746	1	
	rCTP	5 mM Mn^2+^	1.03 ± 0.04	19.9 ± 3.74	5.2 × 10^−2^		2.6 × 10^−2^	
30-mer RNA/50-mer DNA, A	rUTP	5 mM Mg^2+^	0.10 ± 0.01	423 ± 90.4	2.36 × 10^−4^	1		
	rUTP	5 mM Mn^2+^	3.51 ± 0.19	12.8 ± 2.25	2.74 × 10^−1^	1161	1	
	rCTP	5 mM Mn^2+^	1.03 ± 0.07	17.5 ± 4.92	5.9 × 10^−2^		2.2 × 10^−1^	
31-mer RNA/75-mer DNA, 8-oxoG	rCTP	5 mM Mg^2+^	0.034 ± 0.004	974 ± 270	3.52 × 10^−5^	1		
	rCTP	5 mM Mn^2+^	0.275 ± 0.01	1.25 ± 0.28	2.20 × 10^−1^	6286		
13-mer RNA/29-mer DNA, TT dimer	rATP	5 mM Mg^2+^	0.0083 ± 0.001	1678 ± 445	4.94 × 10^−6^	1		
	rATP	5 mM Mn^2+^	0.174 ± 0.005	11.3 ± 1.35	1.54 × 10^−2^	3117		

^a^ Magnitude of stimulaton by Manganese was calculated as relative efficiency: f_rel_ = (k_cat_/K_m_)_Mn^2+^_/(k_cat_/K_m_)_Mg^2+^_. ^b^ Misinsertion frequency was calculated as relative efficiency: f_rel_ = (k_cat_/K_m_)_incorrect_/(k_cat_/K_m_)_correct_. ^c^ Steady-state kinetics: k_cat_ is the turnover rate of the enzyme and K_m_ is the Michaelis–Menten constant. Rows showing data measured in the presence of Mn^2+^ have a blue background.

**Table 6:** Comparison of the effect of Mg^2+^ and Mn^2+^ on the catalytic activity and fidelity of human Polη.

Substrate (Primer/Template), Templating Nucleotide	Incoming Nucleotide	Cation	Velocity Constant	Affinity Constant	Efficiency	Magnitude of Stimulation, Mn^2+^/Mg^2+ a^	Misinsertion Frequency ^b^	Reference
			k_cat_ (min^−1^) ^c^	K_m_ (μM) ^c^	k_cat_/K_m_ (min^−1^ μM^−1^)			[133]
8-mer/11-mer, A	dTTP	5 mM Mg^2+^	109 ± 13	5.4 ± 0.7	20	1		
	dTTP	Mn^2+^	82 ± 5	0.44 ± 0.04	186	9.2		
8-mer/11-mer, cdA	dTTP	5 mM Mg^2+^	8.6 ± 0.5	570 ± 70	0.015	1		
	dTTP	Mn^2+^	10.1 ± 0.2	0.49 ± 0.07	21	1370		
			k_cat_ (10^−3^ s^−1^) ^c^	K_m_ (μM) ^c^	k_cat_/K_m_ (10^−3^ s^−1^ μM^−1^)			[141]
18-mer DNA/25-mer DNA, N7BnG	dCTP	5 mM Mg^2+^	20.6 ± 3.6	10.2 ± 2.4	2.1	1		
	dCTP	1 mM Mn^2+^	38.7 ± 4.4	5.6 ± 0.9	6.9	3.3		
	dTTP	5 mM Mg^2+^	11.5 ± 0.3	51.7 ± 5.3	0.2	1	1.0 × 10^−1^	
	dTTP	1 mM Mn^2+^	17.8 ± 2.1	18.6 ± 1.9	1.0	5	1.4 × 10^−1^	
			k_cat_ (min^−1^) ^c^	K_m_ (µM) ^c^	k_cat_/K_m_ (min^−1^ µM^−1^)			[153]
30-mer RNA/50-mer DNA, G	rCTP	4 mM Mg^2+^	0.86 ± 0.05	1427 ± 202	6.0 × 10^−4^	1		
	rCTP	4 mM Mn^2+^	1.27 ± 0.04	4.9 ± 0.6	2.6 × 10^−1^	430	1	
	rUTP	4 mM Mn^2+^	0.064 ± 0.004	995 ± 226	6.5 × 10^−5^		2.5× 10^−4^	
30-mer RNA/50-mer DNA, C	rGTP	4 mM Mg^2+^	0.34 ± 0.06	6260 ± 1564	5.5 × 10^−5^	1		
	rGTP	4 mM Mn^2+^	0.54 ± 0.02	7.9 ± 0.9	6.9 × 10^−2^	1260	1	
	rUTP	4 mM Mn^2+^	0.030 ± 0.004	1274 ± 519	2.4 × 10^−5^		3.4 × 10^−4^	
30-mer RNA/50-mer DNA, A	rUTP	4 mM Mg^2+^	0.37 ± 0.04	4820 ± 860	7.6 × 10^−5^	1		
	rUTP	4 mM Mn^2+^	0.89 ± 0.03	5 1 ± 2.0	5.9 × 10^−2^	780		
13-mer RNA/29-mer DNA, TT dimer	rATP	4 mM Mg^2+^	0.54 ± 0.04	630 ± 124	8.3 × 10^−4^	1		
	rATP	4 mM Mn^2+^	0.54 ± 0.02	3.6 ± 0.6	1.5 × 10^−1^	180		
31-mer RNA/75-mer DNA, 8-oxoG	rCTP	4 mM Mg^2+^	0.11 ± 0.01	590 ± 123	1.8 × 10^−4^	1		
	rCTP	4 mM Mn^2+^	0.18 ± 0.01	4.0 ± 0.5	4.6 × 10^−2^	260		

^a^ Magnitude of stimulaton by Manganese was calculated as relative efficiency: f_rel_ = k_cat_/K_m_)_Mn^2+^_/(k_cat_/K_m_)_Mg^2+^_. ^b^ Misinsertion frequency was calculated as relative efficiency: f_rel_ = (k_cat_/K_m_)_incorrect_/(k_cat_/K_m_)_correct_. ^c^ Steady-state kinetics: k_cat_ is the turnover rate of the enzyme and K_m_ is the Michaelis–Menten constant. Rows showing data measured in the presence of Mn^2+^ have a blue background.

## Abbreviations

8-oxoG	8-oxoguanine
A	adenine
AP	apurinic/apyrimidinic: (abasic)
ATPase	adenosine-triphosphatase
BER	base excision repair
BPDE	benzo(a)pyrene diol epoxide
C	cytosine
cdA	8:5′-cyclo-2′-deoxyadenosine
CPDs	cyclobutane pyrimidine dimers
dAMP	deoxyadenosine monophosphate
dATP	deoxyadenosine triphosphate
dCMP	deoxycytidine monophosphate
dCTP	deoxycytidine triphosphate
ddNTP	2′3′dideoxyribonucleoside triphosphate
dGMP	deoxyguanosine monophosphate
dGTP	deoxyguanosine triphosphate
DNA	deoxyribonucleic acid
dNMP	deoxyribonucleoside monophosphate
dNTP	deoxyribonucleotide triphosphate
dRP	deoxyribophosphate
DSB	double-strand break
dTMP	deoxythymidine monophosphate
dTTP	deoxythymidine triphosphate
G	guanine
HR	homologous recombination
Mre11	Meiotic Recombination 11 gene
N7BnG	N7-benzylguanine
N7mG	N7-methylguanine
NER	nucleotide excision repair
NHEJ	nonhomologous end-joining
NHMG	N7-nitrogen half-mustard
nt	nucleotide
O6mG	O6-methylguanine
OH	hydoxyl
Pol	DNA polymerase
rAMP	adenosine monophosphate
rATP	adenosine triphosphate
rCMP	cytidine monophosphate
rCTP	cytidine triphosphate
rGMP	guanosine monophosphate
rGTP	guanosine triphosphate
Rev1	Reversionless 1 gene
RNA	ribonucleic acid
rNMP	ribonucleoside monophosphate
rNTP	ribonucleotide triphosphate
rUMP	uridine monophosphate
rUTP	uridine triphosphate
T	thymine
TdT	terminal deoxynucleotidyl transferase
TLS	translesion synthesis
U	uracil
V(D)J	variable: diversity and joining segments of immunoglobulin genes
